# Machine learning-based analysis of a semi-automated PI-RADS v2.1 scoring for prostate cancer

**DOI:** 10.3389/fonc.2022.961985

**Published:** 2022-11-24

**Authors:** Dharmesh Singh, Virendra Kumar, Chandan J. Das, Anup Singh, Amit Mehndiratta

**Affiliations:** ^1^ Centre for Biomedical Engineering, Indian Institute of Technology Delhi, New Delhi, India; ^2^ Department of Nuclear Magnetic Resonance (NMR), All India Institute of Medical Sciences, New Delhi, India; ^3^ Department of Radiodiagnosis, All India Institute of Medical Sciences, New Delhi, India; ^4^ Department of Biomedical Engineering, All India Institute of Medical Sciences, New Delhi, India

**Keywords:** lesion measurement, prostate cancer, prostate imaging-reporting and data system version 2.1, machine learning, MRI

## Abstract

**Background:**

Prostate Imaging-Reporting and Data System version 2.1 (PI-RADS v2.1) was developed to standardize the interpretation of multiparametric MRI (mpMRI) for prostate cancer (PCa) detection. However, a significant inter-reader variability among radiologists has been found in the PI-RADS assessment. The purpose of this study was to evaluate the diagnostic performance of an in-house developed semi-automated model for PI-RADS v2.1 scoring using machine learning methods.

**Methods:**

The study cohort included an MRI dataset of 59 patients (PI-RADS v2.1 score 2 = 18, score 3 = 10, score 4 = 16, and score 5 = 15). The proposed semi-automated model involved prostate gland and zonal segmentation, 3D co-registration, lesion region of interest marking, and lesion measurement. PI-RADS v2.1 scores were assessed based on lesion measurements and compared with the radiologist PI-RADS assessment. Machine learning methods were used to evaluate the diagnostic accuracy of the proposed model by classification of PI-RADS v2.1 scores.

**Results:**

The semi-automated PI-RADS assessment based on the proposed model correctly classified 50 out of 59 patients and showed a significant correlation (*r* = 0.94, p < 0.05) with the radiologist assessment. The proposed model achieved an accuracy of 88.00% ± 0.98% and an area under the receiver-operating characteristic curve (AUC) of 0.94 for score 2 vs. score 3 vs. score 4 vs. score 5 classification and accuracy of 93.20 ± 2.10% and AUC of 0.99 for low score vs. high score classification using fivefold cross-validation.

**Conclusion:**

The proposed semi-automated PI-RADS v2.1 assessment system could minimize the inter-reader variability among radiologists and improve the objectivity of scoring.

## 1 Introduction

Prostate cancer (PCa) is one of the most common cancers in men and the fifth leading cause of cancer-related death globally (1). Over the past several years, multiparametric MRI (mpMRI) has shown the ability to improve the early detection of clinically significant PCa and patient selection for biopsy (2). Prostate Imaging Reporting and Data System version 2.1 (PI-RADS v2.1) was published to simplify the reporting rules, modify imaging sequences, and define clinically significant cancer to reduce the variability in imaging, interpretation, and reporting (3). The PI-RADS assessment system is a qualitative scale with higher values indicating higher suspicion of PCa (3). PI-RADS includes a lesion size-based decision criterion (cutoff = 1.5 cm) to differentiate between score 4 and score 5 and also provides a minimal requirement for the measurement of lesion volume (>0.5 cc for clinically significant PCa) (3). Martorana et al. found that as PI-RADS scores increase, the probability of detecting a clinically significant PCa proportionally increases with increase in lesion volume (4).

Primarily, diffusion-weighted imaging (DWI) in the peripheral zone (PZ) and T2-weighted imaging (T2WI) in the transition zone (TZ) are used to assign the PI-RADS score (3). However, PI-RADS scoring is challenging due to its inherent technical difficulties to visualize a small lesion on MRI, and its subjectivity. Currently, the PI-RADS score is assessed qualitatively by a radiologist, which makes the PI-RADS process time-consuming as reporting time has become an important performance indicator in healthcare (5). Previous studies have shown poor inter-reader variability in the assessment of PI-RADS scores but with the potential to detect clinically significant PCa (6, 7). Artificial intelligence-based workflow systems have shown similar or improved performances in detecting clinically significant PCa compared to radiologists (8) and have the potential to assist radiologists in the screening process by reducing inter-reader variability and evaluation time (9). Recently, one study by Dhinagar et al. (2020) presented a deep learning-based semi-automated model for PI-RADS scoring with limited area under the receiver-operator characteristics curve (AUC) of 0.70 (10). This model was trained and validated to classify lesions with only PI-RADS score 4 and score 5. An automated or semi-automated PI-RADS scoring system for PCa should be able to accurately classify all scores (score 2, score 3, score 4, and score 5) with good accuracy, as each class has a different prognosis for different PI-RADS scores (11). The objectives of this study were **(i)** to develop a semi-automated model for PI-RADS v2.1 scoring in order to speed up and simplify the reporting process and **(ii)** to analyze the diagnostic performance of the proposed model by classifying PI-RADS scores using machine learning methods.

## 2 Materials and methods

### 2.1 MRI data-acquisition

An MRI dataset of 59 men (mean age: 65 ± 8.5 years) with clinically proven PCa (PI-RADS v2.1 score 2 = 16, score 3 = 10, score 4 = 18 and score 5 = 15) was used in this retrospective study with the prior approval from the institutional review board (IRB) of All India Institute of Medical Sciences (AIIMS), New Delhi. Informed consent was waived off by IRB for this study because of the retrospective nature of the study. This study was performed in accordance with institute guidelines and regulations. All prostate MRI examinations were acquired using a 1.5T scanner (Achieva, Philips Health Systems, the Netherlands). T2W images were acquired using a turbo spin-echo sequence with TR/TE = 3330/90 ms, field of view (FOV) = 250×250 mm^2^, reconstructed matrix = 320×320, voxel size = 0.49×0.49×3 mm^3^, slice thickness = 3 mm, slice gap = 3 mm, and number of slices = 36. Diffusion-weighted images were acquired using echo-planar imaging with TR/TE = 6831/81 ms, FOV = 292×292 mm^2^, reconstructed matrix = 112×112, voxel size = 2.6×2.6×3 mm^3^, slice thickness = 3 mm, slice gap = 3 mm, and number of slices = 36, with five b-values of 0, 500, 1000, 1500, and 2000 s/mm^2^. Apparent-diffusion coefficient (ADC) maps were calculated using all five b-values with the least square-optimization to the mono-exponential model using the vendor-provided algorithm at the clinical workstation (12).

### 2.2 Data processing

MRI data in DICOM format were transferred to a workstation (DELL Precision Tower 3620, using Intel^®^ Xeon^®^ CPU E3-1245 v5 @3.50GHz processor and 32GB RAM) and processed using MATLAB^®^ (MathWorks Inc., v2018, Natick, MA). The midgland region of the prostate was used for processing and this region consisted of approximately five to eight slices for each subject. Since most of the prostate cancers (70%–75%) originate in the PZ, this study focused only on this region. In this study, T2WI, DWI, and ADC images were considered for each patient, which was acquired in the MRI examinations.

#### 2.2.1 PI-RADS v2.1 scoring model

The pre-processing steps involved automatic prostate gland and zonal segmentation, 3D image registration, lesion region of interest (ROI) marking, and lesion measurement. The Chan-Vese active contour model along with morphological opening operation was used for prostate gland segmentation and a probabilistic atlas with a partial volume correction algorithm was used to segment the prostate zones into the peripheral zone and transition zone (13). The prostate and its zonal segmentation were performed on the DWI dataset and then the T2W to DWI were registered so that the same segmentation results can be used for both the sequences. An Affine transformation method with a mutual information similarity index was applied for 3D registration of the prostate gland ROIs of T2WI and DWI.

**Figure 1:**
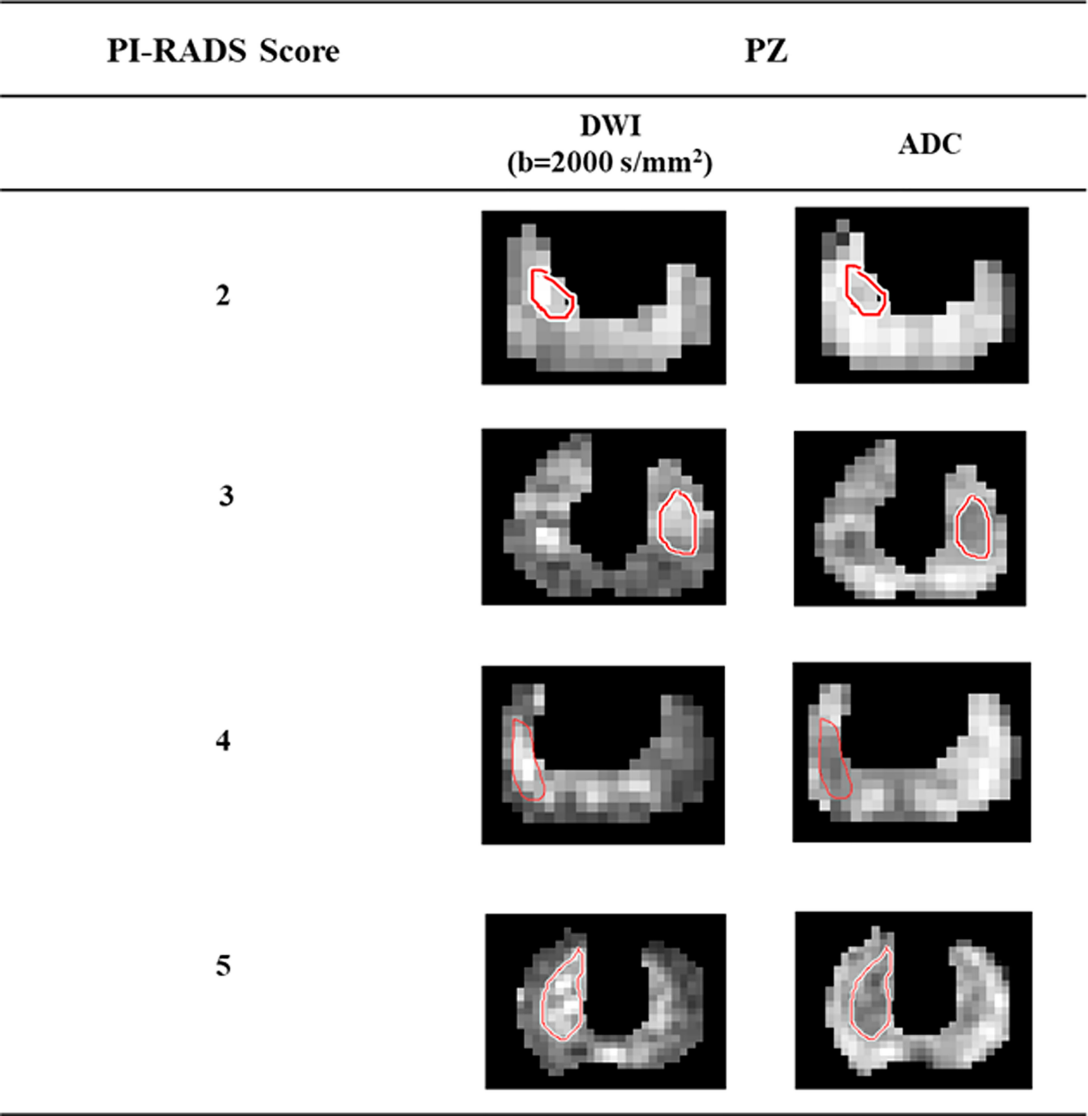
Lesion region of interests delineation from high b-value (b = 2000 s/mm^2^) DWI and ADC of the representative patients with different PI-RADS scores 2 to 5.

All lesion ROIs were manually marked on each slice for all subjects, as per PI-RADS v2.1 guidelines (3) with the help of an expert radiologist (>20 years of experience in prostate imaging). The ROI marking was first demonstrated in a few subjects by the radiologist. The PhD student (or expert) then marked the ROIs, which were verified by the radiologist, and changes to the marked ROIs were made as needed. ROIs were marked on the peripheral zone of DWI (b = 2000 s/mm^2^) data and the same ROIs were used for ADC and registered T2W images of the respective subject. Representative examples of the lesion ROIs for different PI-RADS scores 2 to 5 are shown in **Figure 1**.

**Figure 2:**
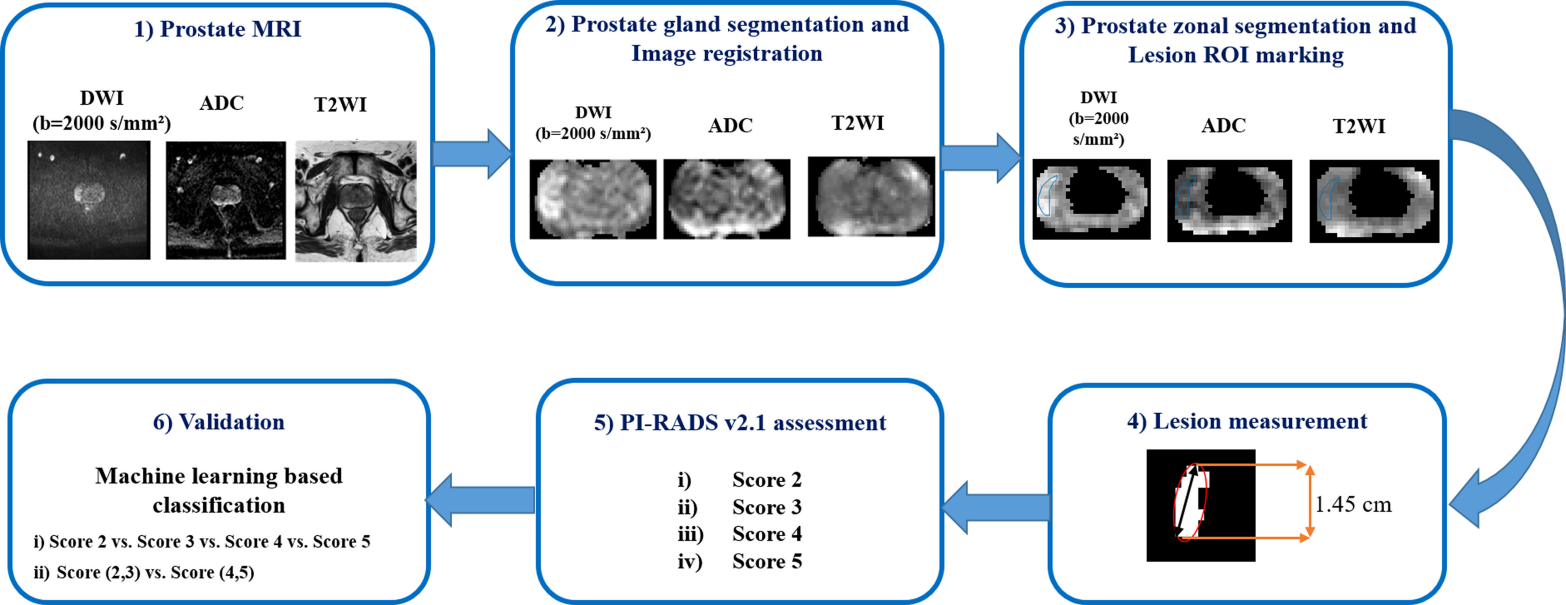
Proposed workflow of the semi-automated model for PI-RARS v2.1 assessment.

PI-RADS v2.1 introduced the lesion maximum diameter and lesion volume-based parameters for evaluating the aggressiveness of lesions. The ellipse fitting-based automatic algorithm was used in this research for the measurement of the lesion maximum diameter and volume, the same as proposed in (14). Maximum diameter was defined as the major axis of the best fitted ellipse. Lesion volume was determined by the multiplication of the slice profile (slice thickness + slice gap) with the summation of all lesion areas in the 2D plane. For comparison, the radiologist manually measured the maximum diameter and volume of the lesion using the image processing software ImageJ (v.1.48; National Institute of Health, Bethesda, USA). In the current study, PI-RADS v2.1 scores were assessed based on the lesion maximum diameter and lesion volume from the fitted ellipse. The workflow of the proposed model for PI-RADS v2.1 assessment is shown in **Figure 2**. The detailed description of all the steps of the proposed model and its parameters are provided in annexure

#### 2.2.2 Performance of the proposed model

Three machine learning methods (linear discriminant analysis (LDA), linear support-vector machine (SVM), and Gaussian SVM) were used to evaluate the diagnostic performance of the proposed model. In this study, the model proposed two different classification approaches: the first approach was to classify PI-RADS scores into four classes: score 2 (n = 16) vs. score 3 (n = 10) vs. score 4 (n = 18) vs. score 5 (n = 15), and the second approach was to classify into two classes: low score (score 2 and score 3) vs. high score (score 4 and score 5).

### 2.3 Statistical analysis

The PI-RADS scores obtained from the proposed model were compared with the radiologist’s assessment using the Pearson correlation coefficient (*r*). Sensitivity, specificity, accuracy, and area under the receiver-operating characteristic curve (AUC) were measured to evaluate the performance of the proposed model. The accuracy of the proposed model was validated using stratified fivefold cross validation.

## 3 Results

### 3.1 Lesion measurement

Lesion maximum diameter and lesion volume measured by the ellipse fitting approach were 0.47 ± 0.06 cm and 0.13 ± 0.03 cc for score 2, 0.67 ± 0.11 cm and 0.29 ± 0.12 cc for score 3, 0.96 ± 0.18 cm and 0.66 ± 0.28 cc for score 4, and 1.45 ± 0.15 cm and 0.99 ± 0.25 cc for score 5. **Table 1** shows the maximum diameter and volume of the lesion measured with manual assessment and automated ellipse fit method for different scores.

**Table 1:** Lesion maximum diameter and volume for different PI-RADS v2.1 scores, in terms of mean ± standard deviation (SD); SD was calculated across all subjects within each score.

Scores	Lesion maximum diameter (cm)		Lesion volume (cc)	
	Manual assessment	Automated Ellipse fit	Manual assessment	Automated Ellipse fit
**Score 2**	0.41 ± 0.08	0.47 ± 0.06	0.10 ± 0.05	0.13 ± 0.03
**Score 3**	0.73 ± 0.12	0.67 ± 0.11	0.36 ± 0.21	0.29 ± 0.12
**Score 4**	1.01 ± 0.19	0.96 ± 0.18	0.74 ± 0.44	0.66 ± 0.28
**Score 5**	1.34 ± 0.24	1.45 ± 0.15	0.93 ± 0.39	0.99 ± 0.25

### 3.2 Semi-automated model-based PI-RADS scoring

**Figure 3:**
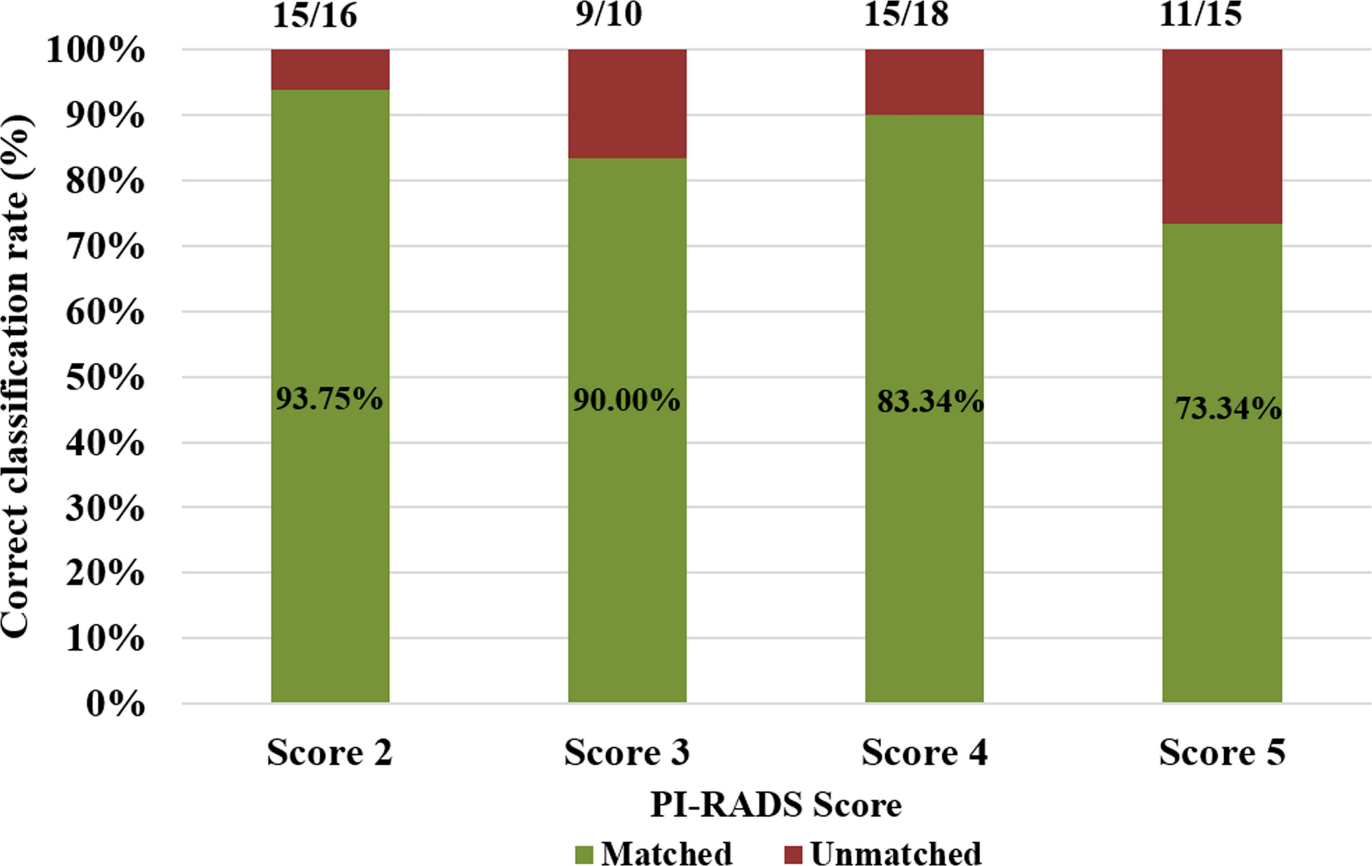
Proportion of the detection rate across all PI-RADS v2.1 scores.

The proposed model-based PI-RADS v2.1 assessment showed 50 out of 59 subjects correctly matched (~85%) with the radiologist assessment. The proportion of the correct classification rate was 93.75% for score 2, 90% for score 3, 83.34% for score 4, and 73.35% for score 5, which are also shown in **Figure 3**. The correct classification rate was also evaluated for the low score (score 2 and score 3) and high score (score 4 and score 5). The correct classification rate was 92.30% for the low score and ~79% for the high score. Semi-automated PI-RADS v2.1 assessment showed a strong positive correlation (*r* = 0.94, p < 0.05) with the radiologist’s assessment.

### 3.3 Diagnostic performance using machine learning methods

**Figure 4:**
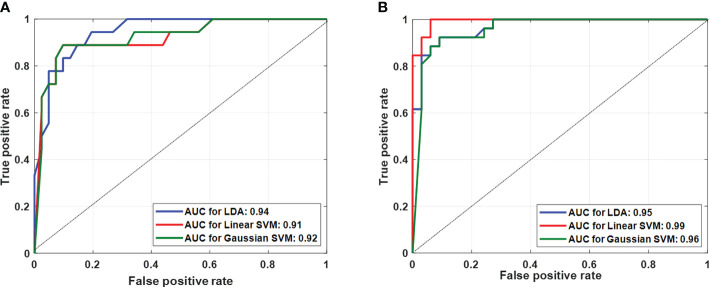
Multiple receiver-operating characteristic graphs for **(A)** score 2 vs. score 3 vs. score 4 vs. score 5 classification and **(B)** low score (2 and 3) vs. high score (4 and 5) classification.

**Table 2** presents the performance of both classifications approaches, **i)** four-class classification (score 2 vs. score 3 vs. score 4 vs. score 5) and **ii)** two-class classification (low score vs. high score) using three different classifiers. The LDA classifier achieved the highest performance with sensitivity of 85.50 ± 1.95%, specificity of 75 ± 1.10%, accuracy of 88.00 ± 0.98%, and AUC of 0.94 in the four-class classification, and linear SVM classifier achieved the highest performance with sensitivity of 91.45 ± 3.65%, specificity of 95.85 ± 1.25%, accuracy of 93.20 ± 2.10%, and AUC of 0.99 in two-class classification using fivefold cross-validation. **Figure 4** shows the ROC graphs for the four-class and two-class classifications using three different classifiers.

**Table 2:** Classification performance of the proposed model **a**) for Score 2 vs. Score 3 vs. Score 4 vs. Score 5 classification using 5-fold cross-validation.

Classifiers	Sensitivity (%)	Specificity (%)	Accuracy (%)	AUC
LDA	85.50 ± 1.95	75.00 ± 1.10	88.00 ± 0.98	0.94
Linear SVM	81.75 ± 2.70	74.30 ± 2.25	83.10 ± 1.10	0.91
Gaussian SVM	86.00 ± 1.50	74.50 ± 1.70	86.40 ± 1.65	0.92
**b)** for Low score (2 and 3) vs. High score (4 and 5) classification using fivefold cross validation				
**Classifiers**	**Sensitivity (%)**	**Specificity (%)**	**Accuracy (%)**	**AUC**
LDA	90.00 ± 2.80	81.25 ± 1.95	89.80 ± 0.95	0.95
Linear SVM	91.45 ± 3.65	95.85 ± 1.25	93.20 ± 2.10	0.99
Gaussian SVM	88.60 ± 2.05	91.67 ± 2.50	86.40 ± 1.90	0.96

## 4 Discussion

The PI-RADS v2.1 has emerged as a technical and reporting standard for uniform interpretation of prostate MRI. However, PI-RADS was challenged by the limited reproducibility among radiologists and medical centers due to its inherent subjectivity in scoring prostate lesions and lack of quantitative metrics (2, 15). An automatic or semi-automatic PI-RADS scoring could assist the radiologist to perform initial screening, speed up reporting, and reduce errors in misclassifying lesions. In this study, a semi-automated PI-RADS v2.1 scoring model was developed for the diagnosis of PCa and validated the diagnostic performance of the proposed model using machine learning methods.

The automatic non-invasive measurement of the prostate lesion could significantly improve to determine the prognosis and assist in PI-RADS assessment. Few approaches for measuring prostate lesions have been reported in earlier studies (16, 17). The maximum diameter and volume of the lesion provide an important information in determining the clinically significant PCa (18, 19). In this study, an automatic ellipse-fitting-based method was used to measure the dimensions of the lesions, and it was found that the results were similar to those obtained from the expert manual measurements. This is because PI-RADS uses only a two-dimensional approach for lesion marking and measurement.

Sanford et al. utilized a convolution neural network (CNN) to evaluate the performance of the automated PI-RADS v2 scoring with an accuracy of 60% for low score vs. high score classification (20). A recent study has presented a semi-automated PI-RADS scoring system using CNN with an AUC of 0.70 (10). However, both studies were limited to a two-class classification only. The proposed semi-automated PI-RADS v2.1 scoring model here outperformed the existing methods with the correct classification rate of 85% and AUC of 0.94 for four-class classification (score 2 vs. score 3 vs. score 4 vs. score 5) and AUC of 0.99 for two-class classification [low score (score 2 and score 3) vs. high score (score 4 and score 5)]. **Table 3** shows a comparison of the performance of the current study with the recent related papers.

**Table 3:** A comparison of the current study’s performance with the recent related articles.

Authors (year)	Number of subjects	Method	Performance
Dhinagar et al. (2020) (10)	617	VGG-16 based deep learning method	ROC-AUC = 0.74 (score 2, 3) vs. (score 4, 5)
Sanford et al. (2019) (20)	196	Convolutional neural network with a ResNet101	Correct classification rate = 60% Sensitivity = 74% for correct depiction of PI-RADS 4/5 lesions
Singh et al. (current study)	59	Image processing and supervised machine learning based model	Overall correct classification rate = 85% accuracy = 88% and AUC = 0.94 for score 2 vs. score 3 vs. score 4 vs. score 5 classification accuracy = 93.20% and AUC 0.99 for (Score 2, 3) vs. (Score 4, 5)

The three supervised classifiers (LDA, linear SVM, and Gaussian SVM) employed in this study are based on the literature, as these three methods are the most commonly used and have been demonstrated to provide better classification performance compared to other classifiers for the prostate MRI (21, 22). These classifiers are fast and have been shown to work well with small datasets (23).

The proposed model for semi-automated PI-RADS v2.1 scoring is relatively objective and could be helpful for non-expert radiologists in terms of reporting accuracy. However, there are some limitations of the study. First, the proposed model is evaluated only for the lesions in the peripheral zone. The second limitation is that the sample size was small. The small sample size in this study provides the preliminary evidence to establish this new automated analysis approach. However a much larger cohort from multiple clinical centers will be essential for wider comparison and clinical acceptability which is beyond the scope of the current manuscript. Third, the reference measurement was done by only one radiologist; inter-observer and intra-observer variability were not evaluated. In this study, it was found that the correct classification rate for scores 4 and 5 is lower than for scores 2 and 3, possibly because the extra prostatic invasion of the lesion was not evaluated and because of inherent curvature bias in ellipse-fitting (24, 25), which needs to be further investigated. An automated lesion ROI marking may improve the model significantly and minimize the workload of radiologist. However, its impact on optimizing clinical workflow in hospital settings was not evaluated, which would require a much larger clinical study.

## 5 Conclusion

The proposed semi-automated model for PI-RADS v2.1 scoring achieved a high classification accuracy of 88% in four-class classification and 93% in two-class classification. This model could reduce the inter-reader variability among radiologists and improve the objectivity of scoring in a screening setting of prostate cancer.

## 6 Annexure

### Step 1: Prostate gland segmentation

A semi-automated method based on a level set formulation of the Mumford-Shah function developed by Chan and Vese was used for segmentation of prostate gland (26). Chan and Vese proposed a pure region-based model to segment image


(1) $$E_{CV}(C,\;c_{1},\;c_{2})=\int_{inside(C)}{\left(u-c_{1}\right)}^{2}dx\;dy\;dz+\int_{outside(C)}{\left(u-c_{2}\right)}^{2}dx\;dy\;dz$$

where, *u* is the segmentation image and *c*_1_ and *c*_2_ are the average intensities of the two regions partitioned by the curve *C*. During the minimization of equation (1), the image is divided into two regions: inside and outside of the curve. The level set framework is combined to minimize the energy function shown in equation (1). The steps of the proposed method for segmentation of the prostate gland using a DWI image (b = 2000s/mm^2^) were as follows: 1) a rectangular mask was constructed manually depending on the prior shape information of the prostate. This mask was further used in all slices of DWI in a subject; 2) a segmentation step to estimate the prostate by Chan-Vese active contour model combining the shape prior, and the range of total iterations was set to 80–100; 3) a refining step to smooth the prostate surface; few surrounding pixels were also found to be labeled as prostate gland. A morphological opening operation (structuring element= disk and size= 2) was applied to remove these speckle pixels. Please refer to (27) for more details of prostate gland segmentation.

### Step 2: Image registration

The prostate gland segmentation was performed on the DWI images (b = 2000s/mm^2^). The segmentation of the prostate gland was also performed individually on the T2W images. The ROIs of T2WI and DWI were used for 3D registration, not the original images. The affine transformation method with a mutual information similarity index was applied for 3D registration.

The affine transformation with 12 degrees of freedom (DoF) allows shearing, scaling, translation, and rotation in three directions. In 3D, affine transformation can be expressed as T: (*x*, *y*, *z*)=

$\left(\begin{array}{ccc} \theta_{11} & \theta_{12} & \theta_{13}\\ \theta_{21} & \theta_{22} & \theta_{23}\\ \theta_{31} & \theta_{32} & \theta_{33} \end{array}\right)\;\left(\begin{array}{c} x\\ y\\ z \end{array}\right)+$


 (2) $$\left(\begin{array}{c} \theta_{14}\\ \theta_{24}\\ \theta_{34} \end{array}\right)$$

where the coefficients parameterize the 12 DoF of the transformation.

### Step 3: Prostate zonal segmentation

This algorithm proposes a novel method for the sub-segmentation of the prostate into peripheral zone and transition zone. The prostate zonal segmentation was performed on the DWI images (b = 2000s/mm^2^). The method is based on probabilistic atlas approach with partial volume (PV) correction algorithm [ii]. Pre-processing steps were carried out for the registration and template creation. Followed by atlas construction, finally, zonal segmentation was performed. The statistical atlas was created by averaging the intensity of images of all training subjects aligned to the image of the target subject based on the corresponding spatial information of PZ and TZ. After that, probabilistic atlases were obtained by counting the number of occurrences of individual pixels in zonal statistical atlases (separate probability map for PZ and TZ) and normalizing the result.

The zonal segmentation of the registered data of the test subject was performed using a probabilistic map at 50% threshold probability (probability= 0.5) for both zones. When binary masks of PZ and TZ were applied to the registered dataset of the test subject, it was observed that some pixels in between the two zones could not be assigned to either of the zones. These pixels were having either equal or less than 0.5 probability values for belongingness to two zones; these pixels constitute the PV zone.

The PV correction algorithm was thus developed for correct assignment of these pixels in the PV zone to either PZ or TZ. For every pixel in the PV zone a belongingness value was calculated separately for both the zones, PZ and TZ, by optimizing the cost function for normalized intensity difference, probability, and Euclidian distance from the corresponding zone. The mask of PZ and TZ was further used in all slices of T2WI and ADC for each subject. Please refer to (27) for more details of prostate zonal segmentation and PV correction steps.

### Step 4: Lesion region of interest (ROI) marking

All lesion ROIs (size range: 50 to 200 voxels) were manually delineated from each slice of all subjects for lesion measurement, based on PI-RADS v2.1 guidelines (3). ROIs were outlined on the PZ of the DWI (b = 2000 s/mm^2^) data with the help of a radiologist (with >20 years of experience in prostate MRI) and the same ROIs were used for the ADC and T2W images of the respective subject.

### Step 5: Lesion measurement

In this study, an automatic ellipse-fitting approach was used for the measurement of lesion maximum diameter and volume. This function employs the least-squares (LS) criterion to estimate the best fit the ellipse to a given set of points (x, y) of the lesion.

The LS estimation is done for the conic representation of an ellipse.

Conic ellipse representation=


 (3) $${\text{a*x}}^{2}{\text{+ b*x*y + c*y}}^{2}+ d*x + e*y +f = 0$$

Maximum diameter was defined as the major axis of the best fit ellipse. Lesion volume was determined by the multiplication of slice profile (slice thickness + slice gap) with the summation of all lesion areas in the two-dimensional plane.

For comparison, the radiologist manually measured the maximal diameter and volume of the lesion using the image processing software ImageJ. (v.1.48; National Institute of Health, Bethesda, MD, USA).

### Step 6: PI-RADS v2.1 assessment

In the current study, PI-RADS v2.1 scores were assessed based on the lesion maximum diameter and lesion volume from the fitted ellipse.

### Step 7: Machine learning based validation

Three machine learning methods (Linear discriminant analysis (LDA), linear support-vector machine (SVM), and Gaussian SVM) were used to evaluate the diagnostic performance of the proposed model. These machine learning methods were implemented using default parameters so as not to introduce a bias or overfit the model on the given data.

LDA is supervised and computes the directions (“linear discriminants”) that will represent the axes that maximize the separation between multiple classes. The LDA approach in detail is shown in (28).

SVM creates a hyper plane in the feature space to divide the data into two classes with the maximum margin. Using a positive semidefinite function, the feature space can map the original features (x, y) into a higher-dimensional space.


 (4) (x, y) → k (x, y)

The function k (·, ·) is called the kernel function. Here, we implemented two standard kernel SVM classifiers.


Linear k (x, y) = (x. y)

Gaussian (5) $$\text{= exp }(\text{- }(∥\text{x - y}∥){\text{/σ}}^{2})$$

where σ is the width of Gaussian. The importance of this parameter relates to cost of constraint violation during the SVM training.

The accuracy of the proposed model was validated using stratified fivefold cross validation.

## Data availability statement

The raw data supporting the conclusions of this article will be made available by the authors, without undue reservation.

## Ethics statement

The studies involving human participants were reviewed and approved by Institutional Review Board, All India Institute of Medical Sciences (AIIMS), New Delhi. The ethics committee waived the requirement of written informed consent for participation.

## Author contributions

DS contributed significantly to the data collection, examination, and analysis and wrote the manuscript. VK contributed to the conception of the study and the revision of the manuscript. CD contributed to the data collection and conception of the study research design. AS contributed to conception of the study and the revision of the manuscript. AM contributed to the conception of the study and the revision of the manuscript and provided constructive discussions. All authors read and approved the final version of the manuscript.

## Funding

The authors would like to acknowledge support staffs of IIT Delhi, New Delhi, and AIIMS Delhi, New Delhi. DS was supported with research fellowship, funded by the Ministry of Human Resource Development, Government of India. This research did not receive any specific grant from funding agencies in the public, commercial or not-for-profit sectors.

## Conflict of interest

The authors declare that the research was conducted in the absence of any commercial or financial relationships that could be construed as a potential conflict of interest.

## Publisher’s note

All claims expressed in this article are solely those of the authors and do not necessarily represent those of their affiliated organizations, or those of the publisher, the editors and the reviewers. Any product that may be evaluated in this article, or claim that may be made by its manufacturer, is not guaranteed or endorsed by the publisher.
